# Experimental tumor growth of canine osteosarcoma cell line on chick embryo chorioallantoic membrane (in vivo studies)

**DOI:** 10.1186/s13028-017-0298-8

**Published:** 2017-05-12

**Authors:** Magdalena Walewska, Izabella Dolka, Anna Małek, Anna Wojtalewicz, Agata Wojtkowska, Artur Żbikowski, Roman Lechowski, Katarzyna Zabielska-Koczywąs

**Affiliations:** 1 Department of Small Animal Diseases with Clinic, Faculty of Veterinary Medicine, Warsaw University of Life Sciences, Nowoursynowska 159c, 02-776 Warsaw, Poland; 2 Department of Pathology and Veterinary Diagnostics, Faculty of Veterinary Medicine, Warsaw University of Life Sciences, Nowoursynowska 159, 02-776 Warsaw, Poland

**Keywords:** Chick embryo, Chorioallantoic membrane, Osteosarcoma, Dogs, Cell line

## Abstract

The chick embryo chorioallantoic membrane (CAM) model is extensively used in human medicine in preclinical oncological studies. The CAM model has several advantages: low cost, simple experimental approach, time saving and following “3R principles”. Research has shown that the human osteosarcoma cell lines U2OS, MMNG-HOS, and SAOS can form tumors on the CAM. In veterinary medicine, this has been described only for feline fibrosarcomas, feline mammary carcinomas and canine osteosarcomas. However, in case of canine osteosarcomas, it has been shown that only non-adherent osteosarcoma stem cells isolated from KTOSA5 and CSKOS cell lines have the ability to form microtumors on the CAM after an incubation period of 5 days, in contrast to adherent KTOSA5 and CSKOS cells. In the presented study, we have proven that the commercial adherent canine osteosarcoma cell line (D-17) can form vascularized tumors on the CAM after the incubation period of 10 days.

## Background

Osteosarcoma is the most common malignant bone tumor in children and dogs, although the disease occurs in dogs approximately 10 times more frequently than in humans. Chemotherapy and aggressive surgical techniques poorly improve the survival time. Consequently, a number of studies on new therapeutics are being conducted. In human medicine, the chick embryo chorioallantoic membrane (CAM) model is extensively used in preclinical oncological studies [1–10]. Growth of various human tumors (Burkitt lymphoma, ovarian cancer, glioblastoma multiforme, head and neck squamous cell carcinoma) was successfully achieved using the CAM model [6, 11–13]. The CAM model is an ideal environment to assess tumor growth, as it is naturally immunodeficient, what is a major advantage compared to using immune-compromised rodent models. Moreover, it follows the “3R principles” (replacement, reduction and refinement) and it is characterized by several other advantages such as easy access, extensive vascularization and a relatively simple experimental approach [5]. The CAM model allows to: expand the knowledge of tumor biology and metastasis [4, 8, 12], perform angiogenesis study [14] and examine the efficiency of anticancer drugs [5]. In veterinary medicine, this model has been described for feline injection-site sarcoma (FISS) [5, 15], feline mammary gland carcinoma [16] and canine osteosarcoma [17]. Pang et al. [17] demonstrated that canine osteosarcoma non-adherent stem cells isolated from KTOSA5 and CSKOS cell lines have higher ability to form microtumors on the CAM than adherent KTOSA5 and CSKOS cells that were dying and were unable to establish growth. As a result, the aim of our study was to assess the ability of commercial canine adherent osteosarcoma cell line (D-17) to form solid tumors on the CAM.

**Fig. 1:**
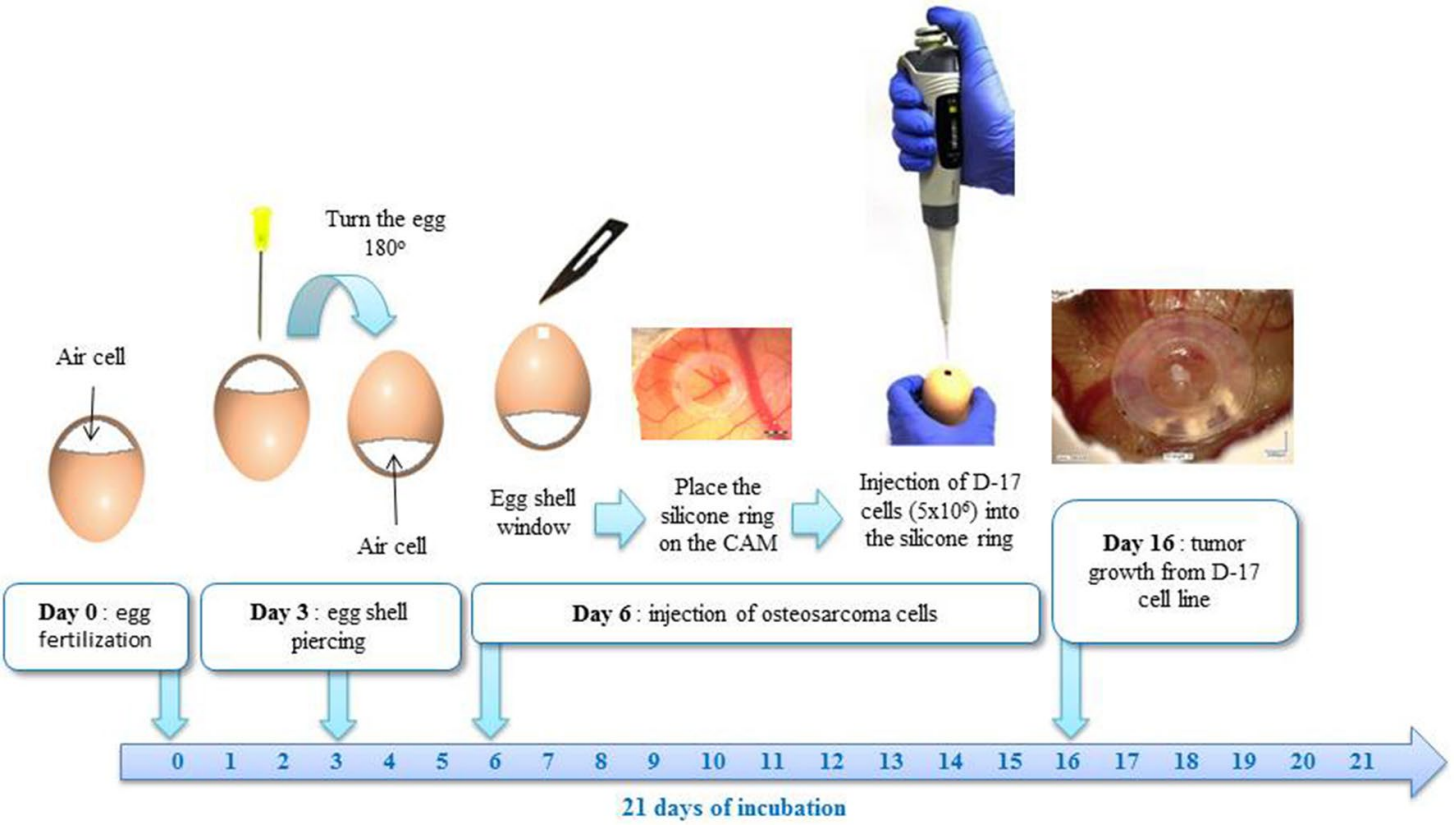
Illustration of the CAM assay indicating critical steps of the experiment

**Fig. 2:**
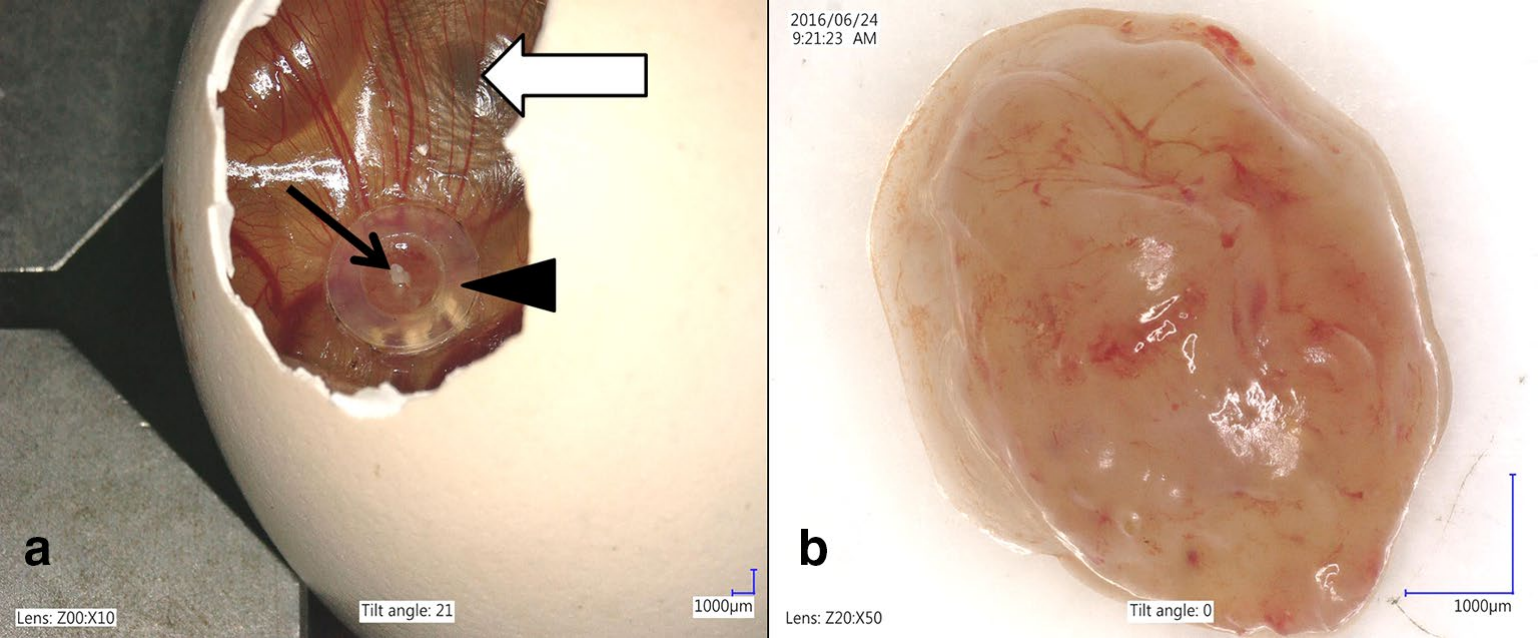
Canine osteosarcoma from D-17 cell line growth on the CAM. **a** Chick embryo (*white arrow*), silicone ring (*arrowhead*), tumor growth (t*hin black arrow*), **b **isolated tumor

**Fig. 3:**
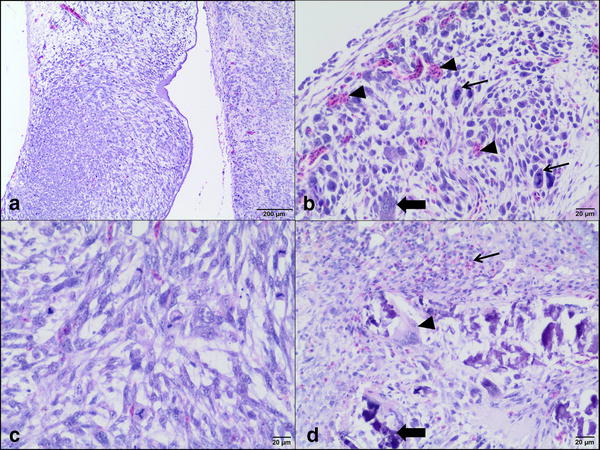
Photomicrographs of canine osteosarcoma growth on the chick embryo chorioallantoic membrane. **a** Overview of tumor growth of canine osteosarcoma cell line. H–E,* bar*: 200 µm; **b** Visible high cellularity of the tumor with pleomorphism, bizarre mitotic figures (*arrows*), multinucleated giant cells (*thick black arrow*), proliferation of small blood vessels (*arrowheads*). H–E,* bar*: 20 µm; **c** tumor mass composed of polygonal to spindle cells separated by fine fibrovascular matrix and showed high mitotic rate. H–E,* bar*: 20 µm; **d** basophilic deposits (*thick black arrow*) surrounded by osteoclast-like giant cells (*arrowhead*) and the infiltration of heterophils (*arrow*). H–E,* bar*: 20 μm

**Fig. 4:**
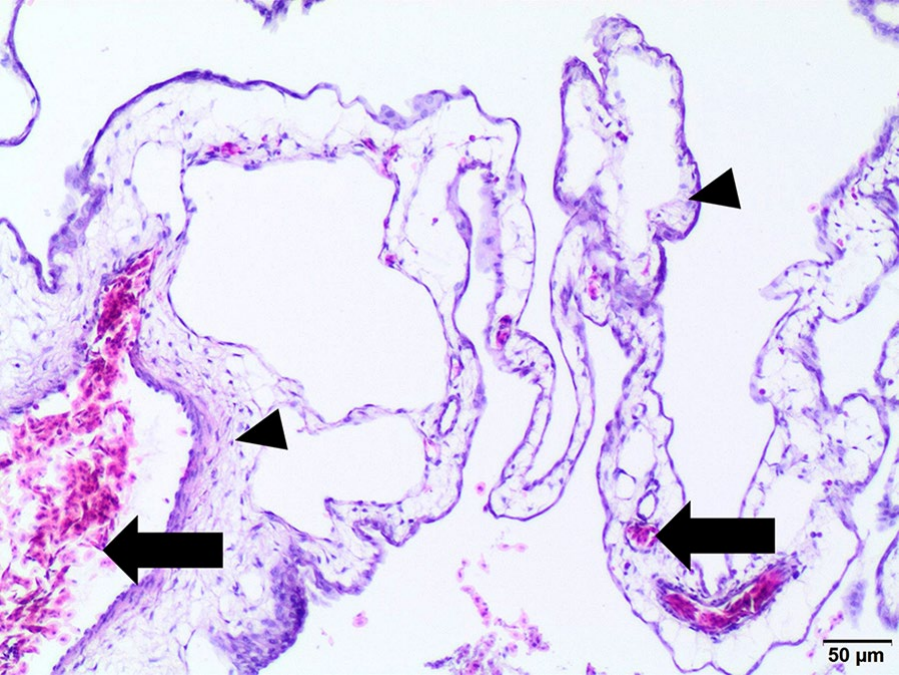
Photomicrograph of the normal chick embryo chorioallantoic membrane. Mild to sparse (*arrowhead*) connective tissue and few blood vessels visible. H–E* bar*: 50 μm

**Fig. 5:**
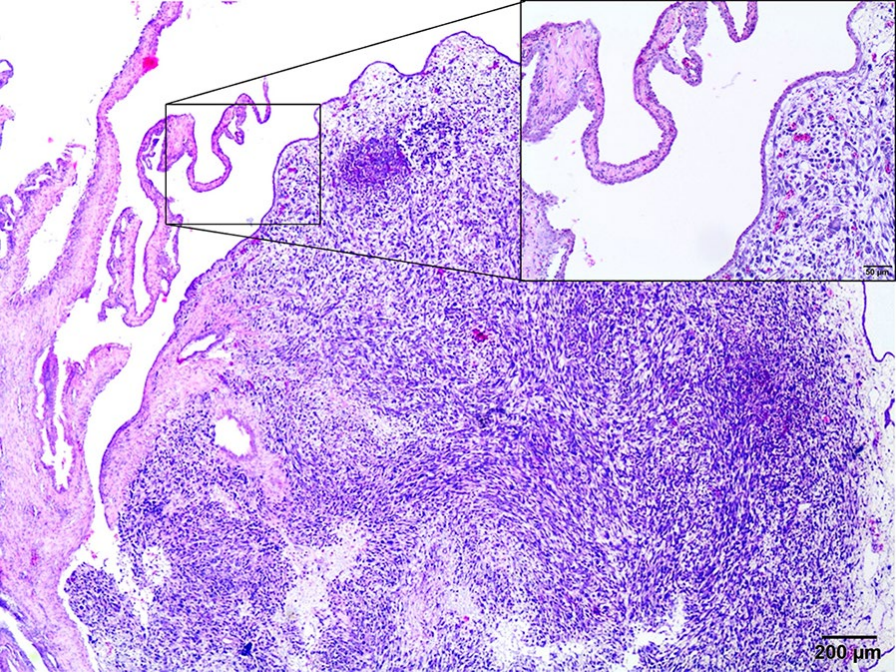
Photomicrographs showing tumor growth from canine osteosarcoma cell line (D-17) on the CAM.* Bar*: 200 μm. High magnification images taken from the sites indicated by* box* comparing CAMs: infiltration of the CAM by tumor cell line (*right*) and showing the normal structure of the CAM (*left*). H–E,* bar*: 50 μm

Commercial canine osteosarcoma cell line (D-17, ATCC) was cultivated in Eagel’s Minimum Essential Medium (EMEM) with the addition of heat-inactivated fetal bovine serum (FBS), penicillin–streptomycin (50 IU ml^−1^) and amphotericin B (2.5 mg ml^−1^) under standard conditions (5% of CO_2_, 95% of humidity, 37 °C). The medium was changed every 48–72 h when 70–80% of confluence was achieved. For performing in ovo study, the cells were trypsinized and counted with Countess II FL Automatic Cell Counter. In ovo assay was performed according to previously described procedure [15] with some modification (Fig. 1). Thirty-six hatching Ross 308 eggs (Poultry Hatchery Pankowski Jan, Białobrzegi, Poland) were held in the incubator and incubated at standard conditions (37 °C, 55% humidity, turn once per hour during the first 6 days). On the 3rd day of incubation, eggs were pierced with a needle (20 G) through the eggshell to the air cell after disinfection of the superficial surface and taped with a semitransparent patch to prevent chick embryos from drying out. Afterwards the eggs were turned 180° to increase the survival rate of chick embryos and to enable easier access to the CAM. On the 6th day of incubation ‘windows’ in the eggshell (sized 7 × 7 mm) were made. After that sterile silicone rings, designed especially for this purpose (7 mm in external diameter, 6 mm in internal diameter, 1 mm thick) (Zegir PTHU, Poland) were placed on the CAM. Osteosarcoma cells (5 × 10^6^ cells per egg suspended in 25 µl of medium) were aseptically injected into the silicone rings. The number of cells seeded per egg were chosen according to the results of our previous study on FISS [5, 15]. Saline and medium (EMEM) (25 µl per egg) were used as negative controls. After inoculation the ‘windows’ were taped with semitransparent patch of high air and humidity permeability. The eggs were candled 24 and 48 h later to evaluate the chick embryos vitality. Tumor growth was visible after incubation period of 10 days. From the 16th day of incubation, the tumor growth was systematically controlled using a digital macro view otoscope (Welch Allyn Viewer, USA) and 3D microscope (VHX-5000, Keyence, Belgium). On the 19th day of incubation, chick embryos were decapitated and the tumors were sampled. The experiment was repeated in triplicate. Tumors were fixed in 10% neutral buffered formalin for 24 h and standard procedures for histopathology were applied. Briefly, tissue samples were embedded in paraffin and cut into 4 μm sections and stained with haematoxylin and eosin (H–E). Histopathological examination and grading were performed according to a system adopted from human medicine and used in previous studies of canine subcutaneous soft tissue sarcomas, in this case for soft-tissue osteosarcomas (extraskeletal) [18]. Histologic tumor grade (low being the 1st, moderate the 2nd and high the 3rd) was assessed with respect to the degree of differentiation, mitotic score and percentage of microscopic tumor necrosis. The mitotic index (MI) was determined in 10 high-power fields (HPF) under the 40× objective lens. Areas with necrosis were omitted. Positive tumor development was recorded when tumors size exceeded 2 mm in diameter according to Balke et al. [1]. Location of the tumor was directly in the silicon rings (Fig. 2a) or a few millimeters out of silicon rings, either the tumor cells had spread through the vessels or the silicon ring position had slightly moved during embryogenesis. Tumors had a smooth surface, an ovoid to spherical shape (Fig. 2b) and they showed an invasive growth pattern into the CAM. The average tumor diameter was 3 mm. Examination of H–E stained tissue sections showed an implant-growth pattern on the CAM (Fig. 3a). The masses were highly vascularized, well demarcated, but non-encapsulated, contained areas of necrosis and haemorrhage. Histologically sarcomas were characterized by high cellularity, abundant mitotic activity (MI 198/10HPF) with the presence of atypical (bizarre) mitoses (Fig. 3b), and lack of osteoid formation in all samples. Tumors were composed of polygonal to spindle cells separated by fine fibrovascular matrix (Fig. 3c). Neoplastic cells showed marked nuclear pleomorphism, round or elongated vesicular nuclei, frequently vacuolated cytoplasm, and prominent eosinophilic nucleoli. There were also scattered multinucleated cells (Fig. 3b). High tumor grade (3rd) was established in all tumors. Peripherally to necrosis, mild to marked mixed cell infiltration were noted (heterophils, mononuclear cells). In one case, some basophilic deposits surrounded by (osteoclast-like) giant cells and heterophils were superficially localized (which were probably a mineral or of uric acid deposits) (Fig. 3d). Angiogenesis, necrosis and hemorrhage were observed as typical features of osteosarcoma. The survival of chick embryos on the 19th day of incubation was 83% (29 out of 35 chick embryos), as six chick embryos died due to manual manipulation. Comparison of the H–E staining of the CAM with and without tumor growth is presented in Figs. 4 and 5.

The experiment proved success of the CAM model for canine D-17 osteosarcoma cell line. In the future, this model may be used to expand the knowledge of canine osteosarcomas. The rodent model, which is commonly used for preclinical evaluation of drug formulation is rather expensive and time-consuming. The in ovo model allows to overcome such disadvantages. The CAM model provides rapid tumor growth at relatively low cost. This model has been successfully adopted in human medicine. Balke et al. [1] reported the ability of three (MNNG-HOS, U2OS, SAOS) out of eight human osteosarcoma cell lines to form vascularized solid tumors on the CAM. In veterinary medicine, the CAM model was firstly reported by our research group for feline vaccine-associated fibrosarcoma cell line (FFS1WAW) [10]. Recently, it has been also described for feline fibrosarcomas (from FFS1 and FFS3 cell lines) [5], feline mammary carcinomas [16] and canine osteosarcomas [17]. Pang et al. [17] demonstrated the ability to form microtumors 5 days after cell inoculation from canine osteosarcoma stem cells isolated from KTOSA5 and CSKOS cell lines, but not from adherent KTOSA5 and CSKOS cells, in contrast to the findings of this study, where we show that the commercial canine adherent osteosarcoma cell line (D-17) can form solid tumors on the CAM (on average 3 mm in diameter). Canine osteosarcoma cell line (D-17) needs 10 days to form solid tumors, which is in agreement with our previous study on feline fibrosarcoma cell line growth on the CAM [15]. However, it is in contrast to the results obtained for various human cell lines, which need only 3–7 days to form solid tumors on the CAM after cell grafting [1, 14]. The lack of tumor growth from adherent canine osteosarcoma cell lines (KTOSA5 and CSKOS) observed by Pang et al. [17], may be due to too short time of observation, as they visualize tumors after 5 days of cell inoculation. Moreover, they injected 10^6^ cells, which may be not enough to obtain tumor growth. Lack of osteoid formation in tumors grown from D-17 cell line may probably result from the short experimental duration (13 days) and the bone was not developed in the early stage of an embryogenesis. It is in agreement with the study performed by Balke et al. [1], who also did not show osteoid formation in tumors growth on the CAM from human osteosarcoma cell lines. The research presented is the first step to create a preclinical oncological model for canine osteosarcoma. Further studies on the CAM model on the ability to metastasize and migrate through the basement membrane of osteosarcoma cells are needed due to rapidly progressive nature of canine osteosarcoma.

## Abbreviations

CAM: chorioallantoic membrane
EMEM: Eagel’s Minimum Essential Medium
FISS: feline injection-site sarcoma
FBS: fetal bovine serum
H–E: haematoxylin and eosin
HPF: high-power fields
MI: mitotic index
